# Retraction Note: Discrimination of urinary exosomes from microvesicles by lipidomics using thin layer liquid chromatography (TLC) coupled with MALDI-TOF mass spectrometry

**DOI:** 10.1038/s41598-022-06491-2

**Published:** 2022-02-07

**Authors:** Nilubon Singhto, Arada Vinaiphat, Visith Thongboonkerd

**Affiliations:** Medical Proteomics Unit, Office for Research and Development, Faculty of Medicine Siriraj Hospital, Mahidol University, Bangkok, 10700 Thailand

Retraction of: *Scientific Reports* 10.1038/s41598-019-50195-z, published online 25 September 2019

The authors have retracted this Article.

After publication it was brought to our attention that there were some technical errors in the identification of individual lipid species; in particular, the mass tolerance was too large, and the ion mode was at times inappropriate for the lipid species. Whilst the data could be re-analysed, this would cause a number of changes to the list of lipid species reported, extending beyond the scope of an Article Correction. We therefore retract this article to prevent any further confusion that our mass spectrometric data may have caused.

All authors agree with this retraction.

